# The effectiveness of faecal removal methods of pasture management to control the cyathostomin burden of donkeys

**DOI:** 10.1186/1756-3305-7-48

**Published:** 2014-01-24

**Authors:** Christopher J Corbett, Sandy Love, Anna Moore, Faith A Burden, Jacqui B Matthews, Matthew J Denwood

**Affiliations:** 1 School of Veterinary Medicine, College of Medical Veterinary and Life Sciences, University of Glasgow, Glasgow G61 1QH, UK; 2 The Donkey Sanctuary, Sidmouth, Devon EX10 0NU, UK; 3 Moredun Research Institute, Pentlands Science Park, Midlothian EP26 0PZ, UK

**Keywords:** Donkeys, Nematodes, Environmental control

## Abstract

**Background:**

The level of anthelmintic resistance within some cyathostomin parasite populations has increased to the level where sole reliance on anthelmintic-based control protocols is not possible. Management-based nematode control methods, including removal of faeces from pasture, are widely recommended for use in association with a reduction in anthelmintic use to reduce selection pressure for drug resistance; however, very little work has been performed to quantitatively assess the effectiveness of such methods.

**Methods:**

We analysed data obtained from 345 donkeys at The Donkey Sanctuary (Devon, UK), managed under three different pasture management techniques, to investigate the effectiveness of faeces removal in strongyle control in equids. The management groups were as follows: no removal of faeces from pasture, manual, twice-weekly removal of faeces from pasture and automatic, twice-weekly removal of faeces from pasture (using a mechanical pasture sweeper). From turn-out onto pasture in May, monthly faecal egg counts were obtained for each donkey and the dataset subjected to an auto regressive moving average model.

**Results:**

There was little to no difference in faecal egg counts between the two methods of faecal removal; both resulted in significantly improved cyathostomin control compared to the results obtained from the donkeys that grazed pasture from which there was no faecal removal.

**Conclusions:**

This study represents a valuable and unique assessment of the effectiveness of the removal of equine faeces from pasture, and provides an evidence base from which to advocate twice-weekly removal of faeces from pasture as an adjunct for equid nematode control. Widespread adoption of this practice could substantially reduce anthelmintic usage, and hence reduce selection pressure for nematode resistance to the currently effective anthelmintic products.

## Background

For the last 50 years, control of nematode parasites of equids in the western world and beyond has focussed on the regular use of anthelmintic compounds [1]. Anthelmintic compounds have been highly effective in controlling large strongyle spp., including the highly pathogenic *Strongylus vulgaris*[2,3], shifting focus towards the previously less problematic smaller strongyles (cyathostomins) [4]. Anthelmintic resistance in cyathostomin species was first detected within a few years of the licensing of these products for use in equids, closely corresponding to the widespread adoption of six to eight-week interval dosing [5] and resistance has developed to each anthelmintic after its introduction [6,7]. Despite a decline in the prevalence of *S. vulgaris* and the introduction of targeted anthelmintic treatment protocols in the 1990s [7], interval dosing with anthelmintics was still common practice in 2002 [8,9]. Targeted threshold dosing involves selecting which animals to dose using faecal worm egg counts (FWEC), typically using an arbitrary lower threshold of 200 eggs per gram (EPG) [2,10]. This strategy is appealing because of the high levels of over-dispersion of nematodes within populations [10,11]. As only a few individual animals theoretically host the majority of the ‘in host’ worm population [12,13], targeted dosing of these animals should only be sufficient to substantially reduce nematode contamination on pasture. Despite a gradual move towards more selective anthelmintic use such as this, the prevalence of anthelmintic resistance in some equine populations is now very high, with resistance now reported to every class available for use in equids [14-16]. The widespread prevalence of anthelmintic resistance in cyathostomins in equids provides strong motivation to develop alternative control methods that do not rely solely on the use of anthelmintic compounds. Commonly adopted practices aim to break the transmission cycle of the parasites by preventing or reducing re-infection of animals through grazing. Methods such as dung removal from pasture [7,17,18], strip grazing and resting pasture have been advocated [19], although few quantitative studies have been undertaken to truly demonstrate their effectiveness.

Under optimum environmental conditions, cyathostomin infective third stage larvae (L3) can develop in faeces within three to four days [20]. In Scotland the shortest interval measured for L3 to translate onto pasture after faecal contamination was two weeks under summer weather conditions [21]. Cyathostomin L3 become markedly less infective after eight days at 38°C [20], exhibiting improved survival at lower temperatures [22]. Dung removal is thought to remove parasite eggs before the first stage larvae (L1) hatch and develop to L3, which then migrate from the faeces onto the pasture [23]. As well as these commonly used control measures outlined above, other methods such as altering diets, and administration of nematophagous fungi have also been investigated for controlling nematodes [24,25]. The wide variety of alternatives to anthelmintic dosing currently being investigated demonstrates the importance of alternative control measures. There is therefore a need for clear scientific evidence to support or contradict the adopted practices. Here, we seek to provide such evidence by examining the effect of removal of faeces from pasture on the individual monthly FWEC of co-grazed donkeys.

## Methods

### Animal management

All donkeys in this study resided at The Donkey Sanctuary, Devon, UK. This is a charity, comprised of seven farms in England that house over 2,500 donkeys at any one time. The animals are managed in distinct groups in separate fields and are generally grouped by age and medical condition, with group sizes ranging from two up to over one hundred. On most pastures, faecal removal is practiced for parasite control; however, on steeper, less accessible fields there is no faecal removal. Helminth control varies slightly between the farms, although the general routine (and the routine used in this study) involves taking faecal samples from every animal at four-weekly intervals, FWEC analysis for strongyle (and other nematode species) eggs and anthelmintic dosing based on varying FWEC thresholds. To ensure that the welfare of the donkeys involved in the study was not compromised, any individuals with a FWEC of 2,000 EPG or over at any sampling received a clinical examination by a veterinary surgeon and where appropriate an immediate dose with pyrantel embonate at a dosage of 19 mg/kg was administered. However, there is known resistance in cyathostomins to this compound at the UK Donkey Sanctuary [26]. After consultation with the University of Glasgow School of Veterinary Medicine ethics committee, this study was exempt from formal ethical approval requirements as it was part of standard husbandry techniques.

### Data collection

FWEC data were obtained from the routine parasite monitoring protocol used at the Donkey Sanctuary to control clinical disease due to parasitism. A total of 345 donkeys (147 females and 198 geldings), split among eleven fields, were used here. Of these animals there was an age range from 1 to 46 years old, where 61% were over 20 years old, 24% between 11 and 20 years old and only 15% between 1 and 10 years old. The animals were managed using three different pasture management strategies according to their respective field; these were, “No Removal”, where no faecal removal took place, “Manual”, where faeces were removed manually (using a dung scoop and/or manual removal with gloves) at least twice a week, and “Automated”, where faeces were removed by an automated pasture sweeper (Terra-Vacc, Sweeper, Terra-Vac Ltd, Suffolk, UK) approximately twice a week when weather conditions allowed. A total of 112 donkeys were on fields where no removal took place (split into three fields of 33, 33 and 42 donkeys), 96 donkeys were on fields where manual faecal collection occurred (split into four fields of 16, 17, 23 and 40 donkeys) and 137 donkeys were on fields where automated faecal collection occurred (split into four fields of 15, 27, 33 and 62 donkeys). Monthly FWEC were analysed using a modified McMaster technique [27] with a sensitivity of 50 EPG. This resulted in 1,885 separate FWEC spread over seven consecutive 28-day periods from the 11^th^ of May to the 28^th^ of October 2010, with a total of 274 missing FWEC observations due to individuals not passing faeces during the sampling window for that month. Faeces were collected immediately after being voided and placed into plastic bags voided of air before being processed at the Sanctuary’s laboratory within 24 hours. One pasture sample was obtained from each field at four-week intervals by collecting 400 evenly spaced “plucks” of grass (the amount pinched between forefinger and thumb) per field. Each sample was processed for L3 extraction using the method described by Jorgensen [28] but with the washing by hand replaced by washing in an electrical washing machine (‘Good ideas mini washing machine’, XPB15-2318, Tensor Marketing Ltd. Darlington).

### Statistical methods

FWEC data were modelled using a latent class first order auto regressive moving average (ARMA) model to describe the seven, consecutive monthly FWEC data obtained from each donkey. The observed egg counts were described using an over-dispersed Poisson distribution, with a log link function. The latent states of the initial observation from each donkey were described using a mixed model with random effects of animal and field, a linear effect of age, and fixed effect of gender. Subsequent observations were modelled as dependent on the latent state corresponding to the previous months observation from that individual, as well as a random effect of sampling point (month), fixed effect of anthelmintic dosing after the previous months sampling (where relevant), and a separate moving average effect for each of the three pasture management types representing the force of re-infection from pasture.

The latent class model was fitted using Bayesian Markov chain Monte Carlo (MCMC) methods, using JAGS [29] run from inside R [30] using the runjags package [31]. Minimally informative priors were used for each parameter, and the model was run to convergence as assessed visually using trace plots for each parameter. Model fit using the deviance information criterion (DIC) [32] was used to determine the best fitting candidate model excluding each of the effects of age and gender.

## Results

**Figure 1:**
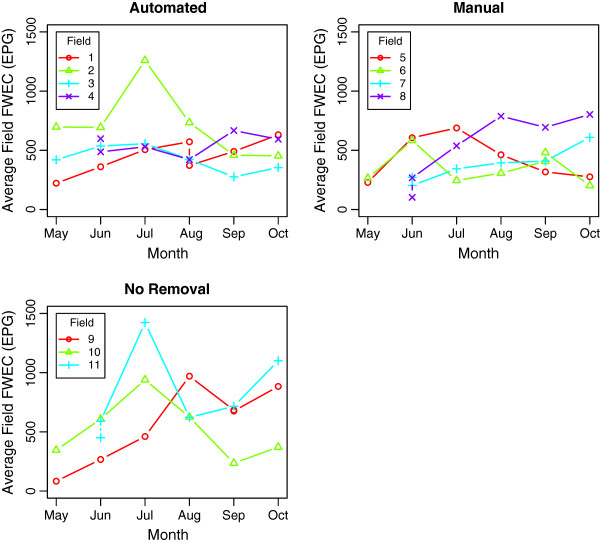
**Mean FWEC of 11 groups of donkeys over a seven-month period between May and October 2010 at The Donkey Sanctuary, Devon.** Groups were managed using either automated or manual faecal removal, or no faecal removal. Fields have been numbered for differentiation.

**Figure 2:**
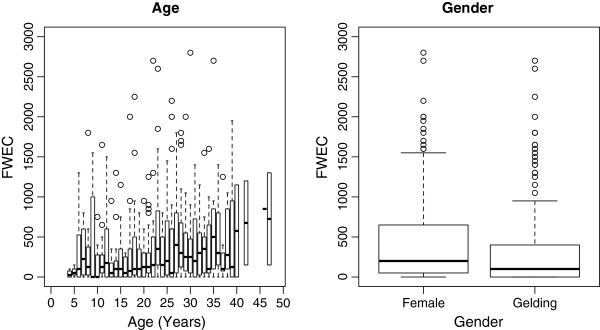
**Box-plot of FWEC by age (left) and gender (right) taken from 310 donkeys in May/June 2010 at The Donkey Sanctuary, Devon.** The boxes represent the 1^st^ and 3^rd^ quartiles with the line in the middle representing the median. The bars represent the maximum result within 1.5 times the interquartile range above and below the 1^st^ and 3^rd^ quartiles respectively, with the dots representing outliers.

**Figure 3:**
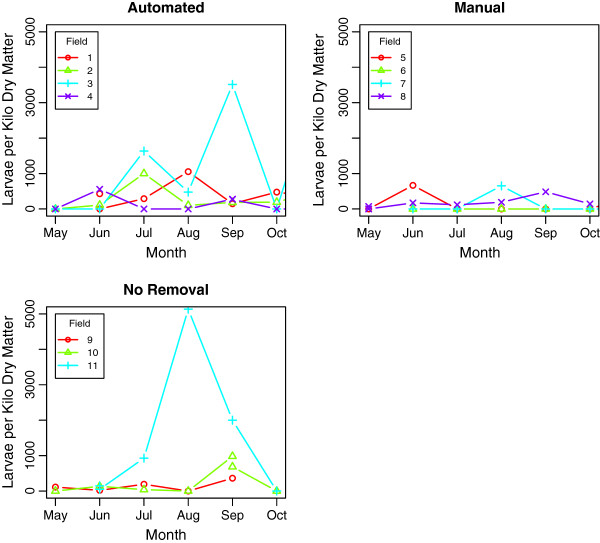
**Pasture larval count of 11 fields measured over a seven-month period between May and October 2010 at The Donkey Sanctuary, Devon.** Groups were managed using either automated or manual faecal removal, or no faecal removal. Fields have been numbered for differentiation.

The individual observed FWEC ranged from 0 to 5,050 EPG. A total of 75 anthelmintic doses were administered to 41 individuals due to the observation of FWEC of 2,000 EPG or greater in samples from these animals. A substantial difference in group mean FWEC was also noted (Figure 1), with a clear summer ‘spike’ in FWEC (with an average of 1075 EPG) observed for the ‘no faecal removal’ fields that was almost absent in 7 of 8 fields where pasture hygiene practices were applied (with a combined average of 629.4 EPG). The observed FWEC patterns did not appear to differ substantially between geldings and females, although a positive relationship between first FWEC sampling and age was observed in the raw data (Figure 2). The pasture larval count for each field, grouped by faecal removal method, is shown in Figure 3. Samples with zero detectable larvae were frequent, particularly in the manual removal groups where 21 of 30 samples recovered no larvae. Pasture larval counts from automated and no removal groups appeared subjectively higher than those from manual removal groups, although this is determined by only a small number of extreme values amongst the majority of much lower (or zero) observations; as a result any statistical analysis of this data beyond descriptive statistics would not be appropriate. Due to a non-parasitic disease outbreak in late May, Field 5 in the manual removal graph of Figures 1 and 3 was changed from automatic to manual faecal removal to help prevent disease transmission. Field 4 from the automated graph in Figures 1 and 3 was changed from manual to automated removal to maintain the number of groups under each management type.

Model fit assessment indicated that the latent class ARMA model of FWEC including both age and gender effects was marginally preferred (with a DIC of 12413.25) compared to the model with an age effect only (DIC of 12413.58), gender effect only (DIC of 12414.34) and neither age nor gender effects (DIC of 12413.47); however, posterior inference for all of the common parameters were very similar between models. Median estimates along with 95% confidence intervals for each of the parameters in the model of best fit are shown in Table 1. The 95% confidence interval for the linear effect of age does not include zero, indicating a significant positive effect of increasing age on individual animal FWEC. No significant effect of gender was found, but, as would be expected, anthelmintic administration had a strongly suppressive effect on subsequent FWEC. The effect of the three pasture management methods relative to each other is also shown in Table 1. There is no evidence for any difference between automated and manual removal, with a median estimate close to zero. However, both automated and manual methods of faecal removal show a suppressive effect on FWEC relative to no faecal removal. The median estimate for both relevant effects was approximately 0.25 on the log scale, corresponding to approximately 20% compound monthly reductions in FWEC where pasture hygiene was applied compared to those fields where faecal removal was not performed.

**Table 1:** Table showing median and 95% confidence intervals for each parameter in the model of best fit

	**Lower95**	**Median**	**Upper95**
Age (years)	0.00194	0.0215	0.0416
Gender (gelding vs female)	-0.652	-0.322	0.0146
Dosing	-3.30	-2.99	-2.68
Automated vs. manual removal	-0.098	-0.010	0.079
Automated vs. no removal	-0.317	-0.234	-0.152
Manual vs. no removal	-0.319	-0.223	-0.130

Median estimate and 95% confidence intervals for each of the parameters included in a latent class ARMA model of 1,885 FWEC obtained from 345 animals over a seven month period between May and October 2010 at The Donkey Sanctuary, Devon.

## Discussion

This study represents the first large-scale quantitative validation of pasture hygiene as a viable method of nematode control in equids. Previous studies have shown faecal removal to be highly effective [18,33,34] but lacked the large sample size and rigorous statistical methods applied here. Our findings demonstrate that removing faeces from pasture approximately twice weekly is an effective adjunct to strongyle control in groups of donkeys. There does not appear to be any difference between manual and automated methods of pasture hygiene in terms of FWEC, suggesting that either method works equally well. This is an important finding because although some pasture sweepers may act to partially disperse the faeces during use, there appears to be no effect on cyathostomin reinfection as measured by FWEC. Although, a qualitative difference was observed between automated and manual removal of faeces as measured by pasture larval counts. The pasture samples were collected in an attempt to quantify the amount of strongyle larvae on the pasture and therefore the relative levels of infectivity. However, inconsistent recovery of L3 as seen in Figure 3 resulting in extreme variability of pasture larval counts within a field precluded statistically meaningful analysis of this data. This suggests that although pasture larval counts are philosophically attractive as providing the most direct measurement of pasture contamination, difficulties relating to the highly variable nature of this data make this technique less precise in practice. With most strongylid larvae found within 30 cm of bovine and equine faeces [35,36], a study in Queensland, Australia focused on free living stages of strongylids of horses and found that migration from the faecal matter is highly influenced by rainfall and temperature [36]. The lack of information about weather conditions and distance from faecal pats of each sample could explain the inconsistent recovery of our pasture larval counts. It must also be noted that as larvae also migrate into the upper layers of the soil, those obtained from the grass only represent a proportion of the total on the pasture [37].

The computationally intensive statistical techniques used in this paper are increasingly being recommended for use in parasitology [38,39]. The long-standing advice for analysis of FWEC data has been to use a generalized linear model (GLM) with an over-dispersed (commonly negative binomial) response [40], because of the potential for erroneous inference associated with log transforming count observations [41]. Bayesian methods have been shown to produce superior inference than older methods of analysis for FWEC data [42] and for analysis of the faecal egg count reduction test (FECRT) data [43]. The MCMC method used here is necessary to fit the latent class model required to describe the complex structure of the data. In particular, the combination of a latent class ARMA model with over-dispersed Poisson response and the additional complication of some missing observations would not have been possible using other modelling frameworks.

With a dosing threshold of 2,000 EPG in this population, the absolute effect of a 20% decrease in egg shedding could be as much as tenfold larger than would be expected in most equine holdings, which use a threshold in the region of 200 EPG. Because of this, and the potential subtle differences in life cycle between donkeys and horses, it is not clear to what extent these conclusions can be extrapolated to other situations; although for parasites with similar lifecycles it would be reasonable to expect a comparable decrease in pasture contamination. Such a decrease would allow less frequent dosing of animals, reducing the amount of anthelmintic used, and therefore slowing the development of anthelmintic resistance. This potentially justifies the expenditure of effort in pasture management in order to reduce the probability of treatment failure due to resistance for clinical cases of parasitism occurring when animals have become heavily parasitised. It is worth noting that FWEC can only be used as an indicator for the number of egg producing adult strongyles. An arbitrary figure of over 1,000 has been described as high [34], but donkeys in this study had FWEC in excess of 5,000 EPG without any observed clinical effects. It is possible that the potential for clinical disease is reduced in donkeys compared to horses.

Although the vast majority of the work on cyathostomins has been undertaken with horses and ponies, it is generally assumed that for the most part these findings apply to all equids providing that the management is similar. The life cycles of the small strongyles are very similar within the two species of equid; encystation of the larval stages is known to occur in the large intestine of horses [5,34] and has also been found in donkeys [44,45]. The reduction of FWEC to zero of donkeys dosed with ivermectin (via intramuscular injection) also appears to be in line with that previously noted in horses [46]. These noted similarities suggest that despite this study being performed in donkeys, the findings and advice could be extended to other equids. If pasture hygiene is combined with strategic anthelmintic dosing, there may be excessive reduction of the cyathostomin population in refugia. This might increase the likelihood of anthelmintic resistance [20] but there is no evidence basis on which we can truly assess the impact of faecal removal on refugia. Certainly, it might be appropriate to increase the arbitrary dosing threshold FWEC of 200 EPG [2,10] in situations when high level parasite hygiene is performed concurrently with an anthelmintic dosing programme.

Evidence of increased FWEC with donkey age suggests a reduced capability to naturally deal with cyathostomins as an animal gets older. Previous findings in younger horses, however, have found FWEC to decrease with age [11], but then increase again with older horses [47]. With only 9 of the 345 donkeys studied under 6 years old (and 85% over 10 years old) these findings are only applicable for middle aged to older donkeys, with a linear effect being less appropriate for a larger age range.

In summary, we have demonstrated and quantified the effectiveness of twice weekly removal of faeces as an adjunct to nematode control in donkeys in a geographical region with a temperate climate and moderate rainfall. These findings support the frequently-given advice to use pasture hygiene for helminth control in equids [17,48], and suggest that a substantial reduction in anthelmintic usage can be achieved. This will result in a reduction in the selection pressure for anthelmintic resistance, potentially prolonging the useful lifespan of the currently effective drugs.

## Conclusions

Twice weekly removal of faecal material from pasture significantly reduced the number of strongyle eggs shed in faeces from groups of co-grazed donkeys. This statistically verifies this form of pasture hygiene as a useful management based control of strongyles in equines. Use of this management control reduces the reliance and use of anthelmintic drugs, reducing the selection pressure towards cyathostomin resistance against these drugs.

## Competing interests

The authors declare that they have no competing interests.

## Authors’ contributions

CJC: Writing, statistics, editing manuscript and read and approved the final version of the manuscript. SL: Experimental design, editing manuscript, and read and approved the final version of the manuscript. AM: Editing manuscript, and read and approved the final version of the manuscript. FAB: Experimental design, overseeing data collection, editing manuscript, and read and approved the final version of the manuscript. JBM: Experimental design, editing manuscript, and read and approved the final version of the manuscript. MJD: Experimental design, statistics, editing manuscript, and read and approved the final version of the manuscript.
